# Extracellular Vesicles: Recent Insights Into the Interaction Between Host and Pathogenic Bacteria

**DOI:** 10.3389/fimmu.2022.840550

**Published:** 2022-05-25

**Authors:** Chaoyu Zou, Yige Zhang, Huan Liu, Yu Wu, Xikun Zhou

**Affiliations:** ^1^State Key Laboratory of Biotherapy and Cancer Center, West China Hospital, Sichuan University and Collaborative Innovation Center for Biotherapy, Chengdu, China; ^2^Department of Hematology and Hematology Research Laboratory, West China Hospital, Sichuan University, Chengdu, China

**Keywords:** extracellular vesicle (EV), bacteria, host, host-bacteria interaction, immune response

## Abstract

Extracellular vesicles (EVs) are nanosized lipid particles released by virtually every living cell. EVs carry bioactive molecules, shuttle from cells to cells and transduce signals, regulating cell growth and metabolism. Pathogenic bacteria can cause serious infections via a wide range of strategies, and host immune systems also develop extremely complex adaptations to counteract bacterial infections. As notable carriers, EVs take part in the interaction between the host and bacteria in several approaches. For host cells, several strategies have been developed to resist bacteria via EVs, including expelling damaged membranes and bacteria, neutralizing toxins, triggering innate immune responses and provoking adaptive immune responses in nearly the whole body. For bacteria, EVs function as vehicles to deliver toxins and contribute to immune escape. Due to their crucial functions, EVs have great application potential in vaccines, diagnosis and treatments. In the present review, we highlight the most recent advances, application potential and remaining challenges in understanding EVs in the interaction between the host and bacteria.

## 1 Introduction

Bacteria are single-celled prokaryotes in which DNA forms circles and is not contained in a defined nucleus. Bacteria are widely distributed around us, and we are interdependent with good (symbiotic) bacteria and fighting against bad (pathogenic) bacteria. Pathogenic bacterial infection is a global threat, and considerable efforts have been made over the years to overcome the diseases. Therefore, with the deepening of research, it has been found that pathogens can change host immune status to avoid immune clearance through various strategies, such as releasing bacterial extracellular vesicles (EVs) (1).

EVs are small compartments surrounded by a lipid bilayer and are unable to replicate (2). “EVs” is a collective term that covers various subtypes, such as exosomes and microvesicles, and both eukaryotic cells and bacteria are able to release EVs (3, 4). In this review, we refer to “hEVs” as EVs from host cells and “bEVs” as bacterial EVs. Resulting from the special generation procedure and structure, EVs contain various bioactive molecules both in the lumen and the surface. Recently, studies have delineated the crucial roles of EVs in biological processes, including influencing metabolism, maintaining homeostasis, and regulating the immune response (4, 5). Observations from several studies have shown that hEVs protect host cells during infection in several ways, such as neutralizing toxins, promoting the release of cytokines and activating adaptive immunity (6, 7). Meanwhile, bacteria release EVs during infection to deliver virulence (8) and escape immune killing (9). However, in some circumstances, hEVs containing bacterial toxins promote disease progression, while bEVs from Lactobacillus reuteri may protect the host from lipopolysaccharide-induced inflammatory responses (10, 11). Thus, how EVs affect the interaction between the host and bacteria has not been fully elucidated.

In this review, we retrieved literature focusing on EVs derived from both host cells and bacteria during infection. We aim to provide an update on recent evidence highlighting new aspects of the role of EVs during infection and discuss the application potential and the challenges facing EV studies.

## 2 Overview of Extracellular Vesicles

### 2.1 EVs From Eukaryotic Cells

Eukaryotic cells constantly and naturally release extracellular vesicles in numerous biological processes. From physiological processes, such as regulating metabolism (12), to pathological processes, such as infection (7) and cancer (13), EVs all play crucial roles. EVs are highly heterogeneous and generated in different pathways. Currently, the generation of the three types of EVs is relatively clear, and they are apoptotic bodies, exosomes and microvesicles. Apoptotic bodies are released from the cell plasma membrane by increased hydrostatic pressure after apoptosis (14). Exosomes form through several processes: (a) invagination of the endosomal membrane; (b) formation of multivesicular bodies; (c) fusion of multivesicular bodies with the plasma membrane; and (d) release from the cell (15). Microvesicles form by a direct outward budding from the cell membrane. Consistent with their generation procedure, EVs are filled with cytosolic proteins such as HSC70, Alix and TSG101 (16). EVs also contain transmembrane proteins such as CD9, CD63 and CD81 (17, 18). Other molecules in the cell matrix, such as RNA, may be encapsulated into EVs during generation, which enables EVs to transfer regulatory messages between cells (19).

### 2.2 EVs From Bacteria

Bacterial EVs (bEVs) were first reported in Gram-negative bacteria in the 1960s (20), while bEVs from Gram-positive bacteria were not reported until 1990 (21). Due to the existence of the thick peptidoglycan cell wall and the lack of periplasmic space and outer membrane in the cell wall, it was thought to be difficult for Gram-positive bacteria to release bEVs; thus, early studies mainly focused on bEVs from Gram-negative bacteria (22, 23). Outer membrane vesicles (OMVs) are well-explored bEVs from Gram-negative bacteria. OMVs bud from the outer layer of the Gram-negative bacterial cell wall and consist of lipopolysaccharide (LPS) and outer-membrane proteins, which are consistent with their cell walls (3). Apart from OMVs, outer-inner membrane vesicles (O-IMVs) are also found in Gram-negative bacteria (24). This kind of bEV contains both outer membranes and cytoplasmic membranes (25) and may be generated by explosive cell lysis (26). Furthermore, bEVs are also detected in Gram-positive bacteria despite their thick cell walls (27). Little is known about the mechanism, and they may be generated by turgor pressure from the cell wall (28) or cell wall­modifying enzymes (29). The last kind of bEV is nanotube, which is tube-like protrusions of the bacterial membrane and functions as bridges between bacteria for substance exchange (30). BEVs play important roles in bacterial biological processes, including transferring DNA (31), killing other bacteria (32), neutralizing phages (33) and transferring virulence to host cells (8).

## 3 Interaction Between Host and Bacteria *via* EVs

### 3.1 HEVs in the Interaction Between Host and Bacteria

**Figure 1:**
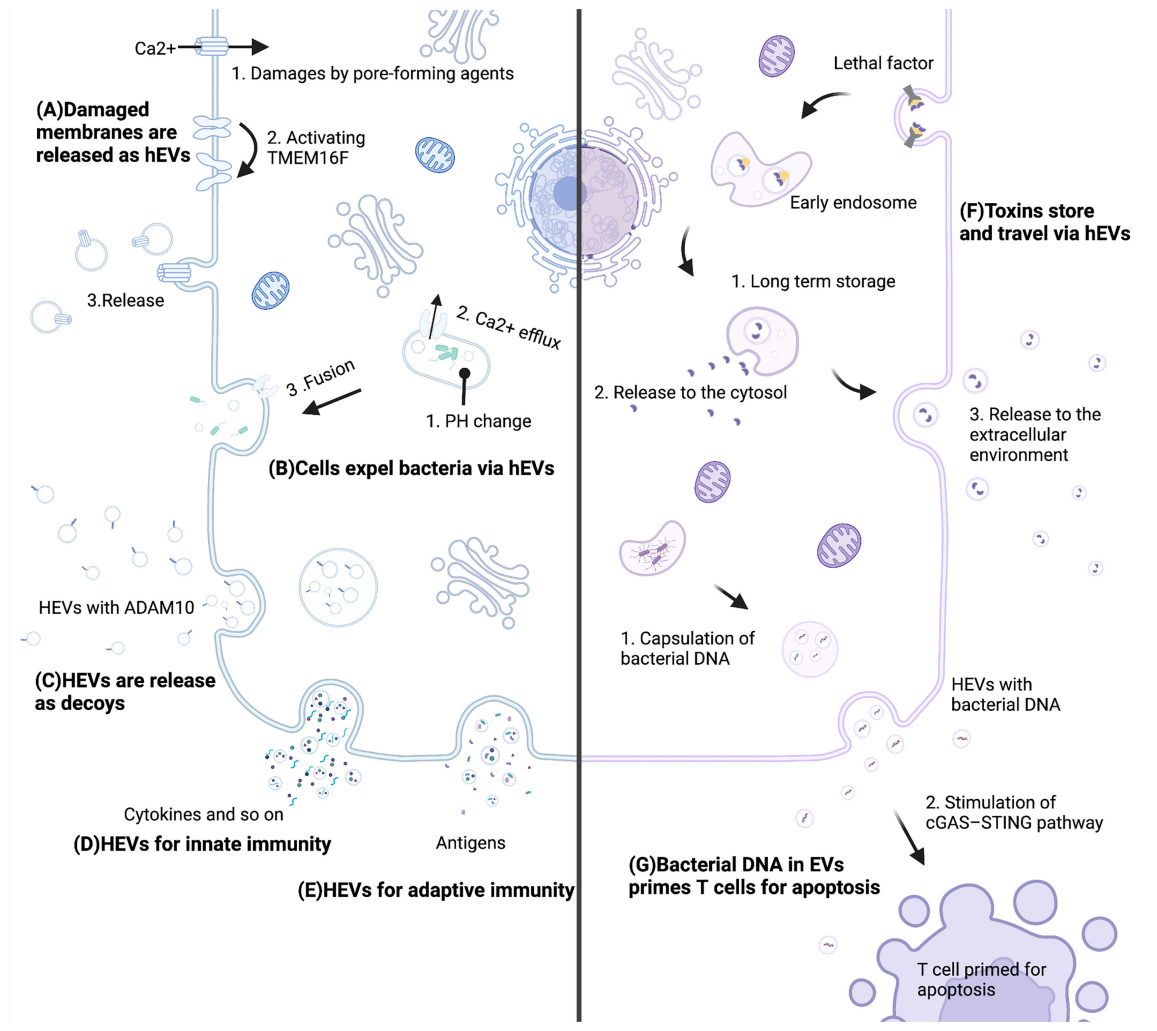
Mechanisms of hEVs in the interaction between the host and bacteria. **(A)** Pore-forming agents lead to Ca2+ influx and activate TMEM16F. Subsequently, damaged membranes are repaired by releasing hEVs. **(B)** A pH change leads to Ca2+ efflux, and this efflux triggers hEVs release to expel bacteria. **(C)** Host cells release ADAM10-containing hEVs to neutralize toxins. **(D)** Cells release molecules such as cytokines *via* hEVs for innate immunity. **(E)** Cells present antigens *via* hEVs for adaptive immunity. **(F)** Lethal factors of *anthrax* store in late endosomes sheltering from degradation and are released to the cytosol and the extracellular environment. **(G)** Bacterial DNA of *Listeria monocytogenes* is encapsulated into hEVs, and these hEVs stimulate the cGAS-STING pathway of T cells, which primes T cells for apoptosis. This figure was created with BioRender.com.

After infection, host-derived EVs (hEVs) demonstrate both antibacterial and probacterial effects under different conditions (7, 34, 35). On the one hand, the host could resist infection through hEVs in several ways. First, damaged membranes induced by bacterial toxins are repaired by releasing hEVs, which is promoted by the calcium-activated lipid scramblase TMEM16F (36) (**Figure 1A**). Second, infected cells expel bacteria through hEVs. When infected with uropathogenic *Escherichia coli*, a lysosomal channel called TRPML3 sensed the change in lysosome pH and led to Ca2+ efflux, and this efflux triggered hEVs release to expel bacteria (37) (**Figure 1B**). Third, hEVs protect host cells from toxins by binding to toxins as decoys (7). In these hEVs, a special protein, named ADAM10 (38), existed on the surface and was able to bind to alpha-toxin of methicillin-resistant *Staphylococcus aureus*, which enabled hEVs to neutralize toxin (**Figure 1C**). Fourth, hEVs participate in innate immunity. Mtb-infected macrophages released hEVs to healthy macrophages, provoking a RIG-I/MAVS/TBK1/IRF3 RNA sensing pathway and the generation of type I interferon (39). HEVs from LPS-activated macrophages induced MyD88/NF-κB signaling and promoted the expression of cytokines and chemokines, such as IL-4, IL-1β, IFN-γ, CCL4 and CCL19 (40) (**Figure 1D**). Fifth, hEVs participate in adaptive immunity by contributing to the T-cell response. Phagocytic events enhanced DCs to release hEVs, and these hEVs demonstrated MHC molecules on the surface, enabling hEV antigen-presenting capacity (6). Infected macrophages were also able to release MHC molecule-demonstrating hEVs for adaptive responses (41) (**Figure 1E**).

On the other hand, pathogenic bacteria attack and damage host cells through hEVs. Lethal factor is one of two parts of anthrax lethal toxin. Lethal factors could be translocated to the lumen of host vesicles during assembly and endocytosis, where they were sheltered from degradation for days and could be delivered to the extracellular medium, leading to damage to the host (10) (**Figure 1F**). Moreover, *Listeria monocytogenes* took advantage of hEVs to deliver its DNA to bystander cells. These bacterial DNA-containing hEVs inhibited T-cell proliferation and induced apoptosis (42). Thus, *Listeria monocytogenes* impaired antimicrobial defense *via* hEVs (**Figure 1G**). In terms of probiotics, the host can release hEVs to modulate probiotic functions. Colonic epithelial cells released hEVs to *Lactobacillus rhamnosus GG*, which might mediate the production of p40. Consequently, *Lactobacillus rhamnosus GG* released p40 into the enteric cavity, protecting epithelial cells against inflammation (35).

### 3.2 BEVs in the Interaction Between Host and Bacteria

Bacterial EVs possess many functions in the interaction between the host and the bacteria. One of the most important functions is to transfer virulence to the host cells. For example, VacA, a *Helicobacter pylori* (*H. pylori)* toxin, was constitutively released *via* bEVs, accounting for 25% of total VacA (43). This bEV-associated VacA was internalized by gastric epithelial cells, which promoted low-grade gastritis to support the persistence of *H. pylori* (44). Bacteria exploit bEVs as an alternative secretory pathway to cover the shortage of other secretory systems, and the mechanisms by which bEV-associated toxins enter host cells may be completely different (45). Compared with free toxin, bEVs provide protection against digestion by intestinal proteases, thus allowing the bacteria to target host cells distant from the primary colonization sites (46). BEVs are able to travel through barriers. In an *in vitro* intestinal epithelial cell model, bEVs could be transported across a polarized cell monolayer with an estimated average uptake efficiency of 30%, suggesting the possibility that bEVs might travel across the gastrointestinal epithelium (47). Moreover, studies showed that bEVs were successfully delivered to the brain crossing the blood–brain barrier and were taken up by meningeal macrophages in mice (48, 49).

Apart from virulence transfer, bEVs contribute to the immune evasion of bacteria. Bacteria release bEVs as strategies to resist antimicrobial molecules from the host. BEVs from *Salmonella* expressed PagC, which was able to bind to complement component C3b and complement inhibitor factor H. These PagC+ bEVs recruited complement inhibitor factor H and degraded active C3 (50). KatA in *H. pylori* EVs detoxified reactive oxygen species released by immune cells and rescued bacteria from killing during the respiratory burst (51). BEVs are also released to resist polymyxin (52) and antimicrobial fatty acids (53), protecting bacteria from harm.

## 4 EVs in Specific Systems and Diseases

Each host system has different strategies and characteristics to address the invasion of pathogenic bacteria. The latest studies have highlighted EV-mediated interactions in several systems, such as the respiratory, gastrointestinal and urinary systems (**Table 1**).

**Table 1:** Roles of EVs in specific systems and diseases.

Roles of EVs in specific systems and diseases					
System	Type of EVs	Bacterium	EV origin	Role/function of EVs	Reference
Respiratory system	Host-derived EVs	*Mycobacterium tuberculosis*	Macrophage	Changes of EV contents: miRNA and mRNA	(54)
		*Mycobacterium bovis bacillus calmette-guerin*	Macrophage	Changes of EV contents: miRNA	(55)
		*Mycobacterium tuberculosis*	Macrophage	Transfer of bacterial RNA	(39)
		*Mycobacterium tuberculosis*	Monocytic cell (THP-1)	Changes of EV contents: proteins	(56)
		*Mycobacterium tuberculosis*	Macrophage (J774 cell)	Changes of EV contents: proteins	(57)
		*Mycobacterium tuberculosis*	Macrophage (RAW264.7 cell)	Participation in innate immune	(58)
		*Mycobacterium tuberculosis*	Macrophage	Participation in innate and adaptive immune	(59)
		*Pseudomonas aeruginosa*	Airway epithelial cell	Participation in innate immune	(60)
	Bacterial EVs	*Mycobacterium tuberculosis*	*Mycobacterium tuberculosis*	Virulence release during intracellular stay	(61)
		*Mycobacterium tuberculosis*	*Mycobacterium tuberculosis*	Pathogenic role during tuberculosis	(62)
		*Pseudomonas aeruginosa*	*Pseudomonas aeruginosa*	Triggering innate immune response	(63)
		*Pseudomonas aeruginosa*	*Pseudomonas aeruginosa*	Triggering innate immune response	(64)
		*Pseudomonas aeruginosa*	*Pseudomonas aeruginosa*	Triggering innate immune response	(65)
		*Pseudomonas aeruginosa*	*Pseudomonas aeruginosa*	Regulating regulatory T cells	(66)
		*Pseudomonas aeruginosa*	*Pseudomonas aeruginosa*	Regulating regulatory T cells	(67)
Gastrointestinal system	Host-derived EVs	*Helicobacter pylori*	Serum	Impairment in endothelial functions	(68)
		*Helicobacter pylori*	Gastric epithelial cell	Impairment in endothelial functions	(69)
		*Helicobacter pylori*	Blood	Impairment in endothelial functions	(70)
		*Helicobacter pylori*	Gastric cancer cell	Contribution to carcinogenesis	(71)
		*Salmonella typhimurium*	Macrophage	Proinflammatory effects	(72)
		*Salmonella typhimurium*	Macrophage	Participation in adaptive immune system	(73)
	Bacterial EVs	*Helicobacter pylori*	*Helicobacter pylori*	Proinflammatory effects	(44)
		*Helicobacter pylori*	*Helicobacter pylori*	Suppression on T cells	(74)
		*Helicobacter pylori*	*Helicobacter pylori*	Contribution to carcinogenesis	(75)
		*Helicobacter pylori*	*Helicobacter pylori*	Impairment in endothelial functions	(76)
		*Salmonella typhimurium*	*Salmonella typhimurium*	Virulence release during intracellular stay	(77)
		*Salmonella typhimurium*	*Salmonella typhimurium*	Virulence delivery	(78)
		*Salmonella typhimurium*	*Salmonella typhimurium*	Triggering autophagy	(79)
Urinary system	Host-derived EVs	Uropathogenic *Escherichia coli*	Bladder epithelial cell	Participation in innate immune system	(80)
		Uropathogenic *Escherichia coli*	Urothelial cell	Damage to the barrier	(81)
	Bacterial EVs	Uropathogenic *Escherichia coli*	Uropathogenic *Escherichia coli*	Virulence delivery	(82)
		Uropathogenic *Escherichia coli*	Uropathogenic *Escherichia coli*	Suppression on inflammation	(83)

### 4.1 Respiratory System

The respiratory system is one of the most investigated systems in terms of EVs. The main topics involve mycobacterial species such as *Bacillus Calmette-Guerin* (BCG), *Mycobacterium tuberculosis* (Mtb) and *Pseudomonas aeruginosa* (*P. aeruginosa*).

#### 4.1.1 HEVs in the Respiratory System

Studies have analyzed exosomal RNA from Mtb-infected or BCG-infected macrophages *in vitro* and revealed that Mtb infection led to changes in RNA in hEVs, including transcripts that were involved in the antibacterial response and miRNAs that might be associated with metabolism and energy production (54, 55). Interestingly, mycobacterial transcripts were also found in hEVs derived from macrophages infected *in vitro* by Mtb (54), and Mtb RNA might be encapsulated through a SecA2-dependent pathway (39). For hEV proteins, 68 proteins originating from hEVs that were released by Mtb-infected macrophages were significantly different compared with the control and were mainly involved in vesicular formation, antigen processing and immune function (56). Forty-one mycobacterial proteins were also identified, and these proteins were highly immunogenic, suggesting a proinflammatory function (57). These changes in the constituents of hEVs usually enhance innate and adaptive immune responses. HEVs from Mtb-infected cells contributed to the release of cytokines and recruitment of host cells (58). HEVs containing mycobacterial antigens were able to activate the adaptive immune response. Subsequently, Mtb-specific CD4+ T cells proliferated (59).

*Pseudomonas aeruginosa* is a common opportunistic pathogen in the lung. After exposure to *P. aeruginosa*, airway epithelial cells released hEVs to macrophages, and these stimulated macrophages released more cytokines and increased the expression of innate immune genes, suggesting an EV-mediated mechanism between bacteria-infected cells and immune cells during infection (60).

#### 4.1.2 BEVs in the Respiratory System

Several mycobacterial species, such as the medically important BCG and Mtb, all possess the ability to release membrane vesicles despite their thick cell walls (28). When Mtb infected macrophages, the macrophages released two distinct EVs: one enriched in host cell markers and the other enriched in Mtb molecules. The release of the latter one was dependent on bacterial viability, which implied that Mtb might release bacterial vesicles during the intracellular stay (61). Mice from the group that were exposed to Mtb EVs first and then infected with Mtb showed accelerated local inflammation, and increased bacterial replication was seen in the lungs and spleens, suggesting a pathogenic role of bEVs during tuberculosis (62).

BEVs from *P. aeruginosa* are complex entities composed of flagellin, lipopolysaccharide and other proteins and are able to induce an inflammatory response in host cells. When mice were exposed to bEVs from *P. aeruginosa*, neutrophils and macrophages accumulated, and multiple cytokines increased, including CXCL1, CCL2, IL-1β, TNF-α, IL-6, and IFN-γ (63). This innate immune response is elicited in several pathways. Both toll-like receptor (TLR)2 and TLR4 knockout mice showed a slight reduction in inflammatory responses, suggesting the involvement of toll-like receptors in the response (63). Apart from toll-like receptors, peptidoglycan in bEVs is recognized by NOD-like receptors in the cytoplasm (64). Inflammasomes, including NLRP3 and NLRC4, are detected during infection (64), and flagellin in bEVs may be a key ligand for NLRC4 inflammasome activation (65). However, in some diseases, bEVs may benefit the host by regulating regulatory T cells. In asthma, bEVs increased the regulatory T-cell response and decreased the Th2 response, which led to the inhibition of airway hyperresponsiveness, inflammation and serum IgE secretion and offered protection against allergic sensitization (66). In ischemia–reperfusion injury, bEV preconditioning attenuated tissue injury and reduced the total protein concentration. This protective effect was achieved by regulating the balance of regulatory T cells and Th17 cells through the Tim-3 and TLR4/NF-κB pathways (67).

### 4.2 Gastrointestinal System

In terms of the gastrointestinal system, the focus of EV studies relies on *H. pylori* and *Salmonella typhimurium*.

#### 4.2.1 HEVs in the Gastrointestinal System

HEVs released by *H. pylori*-infected cells in the stomach can reach the blood circulation and directly affect the cardiovascular system. It was reported that in *H. pylori*-infected patient serum, hEVs contained cagA, a major virulence factor of *H. pylori*. These hEVs promoted foam cell formation and aggravated atherosclerosis (68, 69). Increased exosomal miR-25 was found in *H. pylori*-infected patient blood and was associated with negative cardiovascular events (70). Intriguingly, in addition to negative effects on vascular endothelial cells, a study implied that hEVs from *H. pylori*-infected cells demonstrated protumor effects. *H. pylori*-infected gastric cancer cells released hEVs that contained mesenchymal‐epithelial transition factor and educated macrophages toward a protumorigenesis phenotype (71).

HEVs from *S. Typhimurium*-infected macrophages affect innate and adaptive immunity. For innate immunity, one subtype (CD63+ and CD9+) of infected macrophage-derived EVs triggered the secretion of TLR4-dependent tumor necrosis factor alpha and other cytokines, such as RANTES, GM-CSF, and G-CSF (72). Regarding adaptive immunity, another subtype of hEVs was composed of bacterial antigens, and these hEVs promoted T-helper 1-cell activation and anti-*S. Typhimurium* antibody production (73).

#### 4.2.2 BEVs in the Gastrointestinal System

Quantitative proteomic analyses revealed that bEVs from *H. pylori* contained more than 400 proteins, including the typical virulence factors of *H. pylori*, such as vacA and cagA (8). This constitutively shed bEVs play a role in promoting the low-grade gastritis associated during *H. pylori* infection (44). Toxins in bEVs also suppress T-cell proliferation. This effect is not through a direct effect on T cells but results from the induction of COX-2 expression in monocytes (74). Apart from inflammation, cancer signaling pathway alterations have been identified, including metabolic pathways and the PI3K-Akt signaling pathway (8). VacA in bEVs might contribute to carcinogenesis during *H. pylori* infection (75). Moreover, CagA and LPS from bEVs might injure the endothelium and promote atherosclerotic plaque formation. These processes might be associated with the activation of the ROS/NF-κB signaling pathway (76).

*Salmonella enterica serovar Typhimurium* is an intracellular bacterium and is able to survive in macrophages. To promote bacterial survival and spread, strategies have evolved, and releasing bEVs during the intracellular stay is one of these strategies. After infection with *Salmonella Typhimurium*, epithelial cells released bEV-like vesicles. The release of this vesicle depended on intact *Salmonella*-containing vacuoles in infected cells. This vesicle damaged bystander cells through several processes: (a) anterograde transport on microtubule and actin tracks and release from infected host cells; (b) internalization by bystander cells through active endocytosis; and (c) retrograde transport through the Golgi complex and causing cell damages (77). New virulence factors, including PagK, PagJ, and STM2585A, were also found in bEVs (78). However, the host also develops mechanisms to defend against these attacks. AMPK in host cells was able to recognize bEVs and subsequently activate the autophagy pathway before bacterial invasion (79).

### 4.3 Urinary System

#### 4.3.1 HEVs in the Gastrointestinal System

Uropathogenic *Escherichia coli* (UPEC) is a causative agent in urinary tract infection. During infection, HEVs show both protective and harmful effects. EVs from bladder epithelial cells were enriched in the iron-binding glycoprotein lactoferrin, and this type of hEV reduced bacterial adherence and promoted neutrophil functions (80). However, EVs from urothelial cells in a pyroptotic state led to barrier damage. These hEVs were full of IL-1beta and IL-18, which recruited mast cells. As a consequence, mast cells released tryptase and damaged barrier function *via* the tryptase-PAR2 axis (81).

#### 4.3.2 BEVs in the Gastrointestinal System

UPEC produces a toxin called cytotoxic necrotizing factor type 1 (CNF1). This toxin constitutively activates small GTPases of the Rho family and results in decreased membrane fluidity in mouse polymorphonuclear leukocytes (84). CNF1 was detected in UPEC OMVs, and *in vitro* experiments confirmed CNF1 delivery into host cells by OMVs (82). Further study showed that CNF1-containing OMVs were transferred to polymorphonuclear leukocytes, negatively affecting the efficacy of the acute inflammatory response to these organisms (83).

## 5 Applications

**Figure 2:**
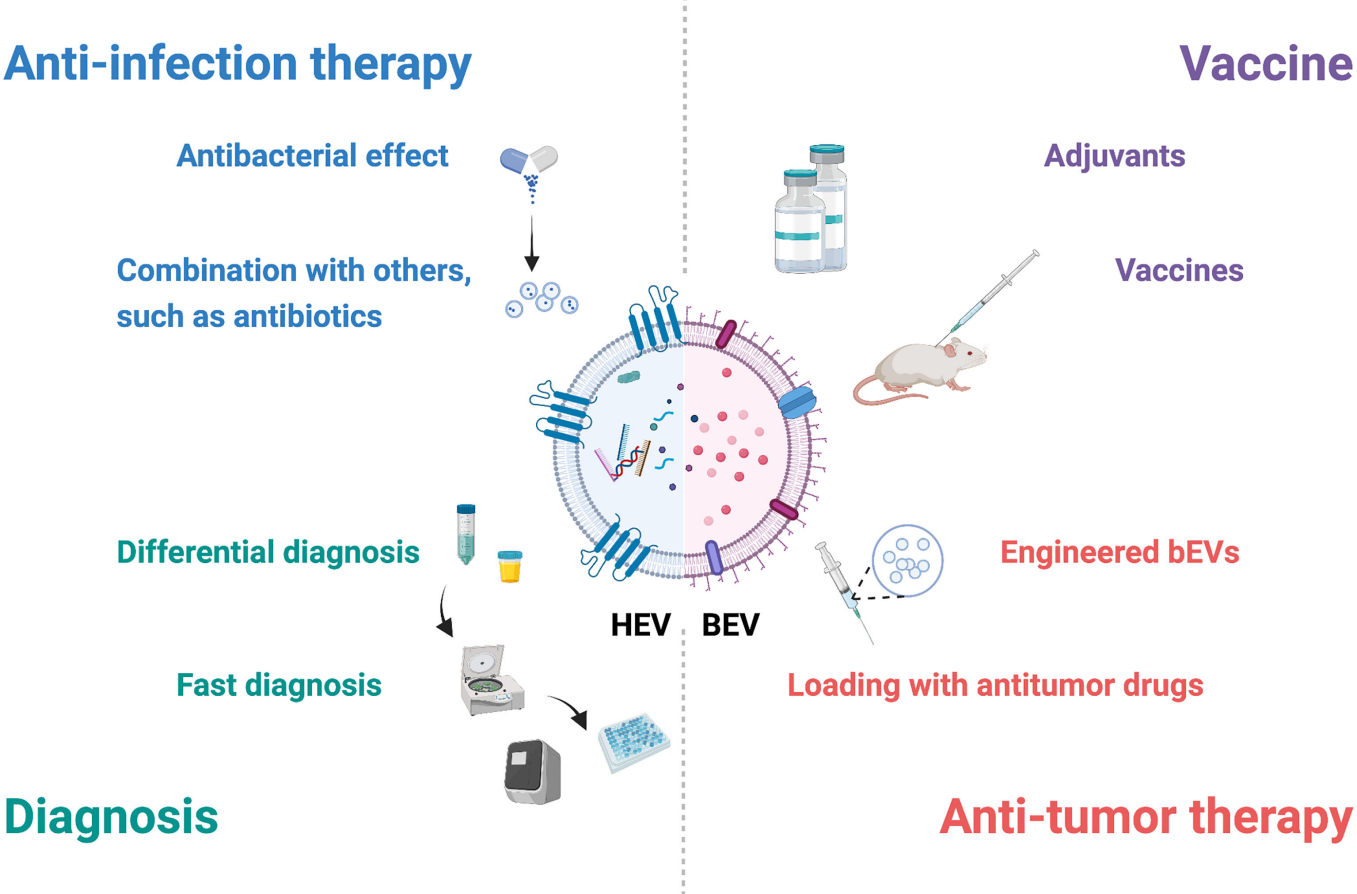
Potential applications of EVs in interactions between the host and bacteria. EVs have potential in diagnosis, anti-infection therapy, vaccines and antitumor therapy. Combined with specific molecules and other techniques, hEVs may be utilized for fast diagnosis and differential diagnosis. HEVs from specific cells, such as mesenchymal stem cells, possess antibacterial effects, and hEVs combined with other materials may show better therapeutic effects. BEVs contain many pathogen-associated molecular patterns that provoke innate and adaptive immune responses and are suitable for adjuvants and vaccines. Engineered bEVs express specific proteins that demonstrate antitumor effects. Meanwhile, these bEVs are able to load antitumor drugs, showing potential in antitumor therapy. This figure was created with BioRender.com.

As crucial participators in the interaction between the host and pathogenic bacteria, growing evidence suggests that EVs have great potential in vaccines, diagnosis and treatment (**Figure 2**).

### 5.1 HEVs for Applications

HEVs carry specific molecules in both the surface and lumen, and these biomarkers differ according to the sources and diseases, which shows the potential for diagnosis. Recognition of biomarkers in hEVs with a fast examination technique provides better and faster results in diagnosis. For example, the combination of hEV DNA and droplet digital PCR presented higher sensitivity for tuberculosis detection, especially when bacterial loads were low (85). HEVs can also be used in differential diagnosis. The ability to aggregate with bacteria ensured hEVs to distinguish bacterial infection from noninfectious inflammation (86). Differences in akt and CD9 from urinary EVs might be markers for differentiating urinary tract infection from asymptomatic bacteriuria (87).

Specific subtypes of hEVs exert therapeutic effects. MSC-derived EVs protected against infection in acute lung injury (88). Combination with other materials, such as antibiotics and other nanomaterials, also improves the therapeutic effects. Either administrating hEVs with antibiotics or using antibiotic-containing hEVs provided better outcomes in infected mice (89). A dressing with a combination of silver nanoparticles and hEVs promoted angiogenesis and wound healing in *P. aeruginosa*-infected mice (90).

### 5.2 BEVs for Applications

BEVs contain many pathogen-associated molecular patterns, such as flagella, peptidoglycans and LPS, which not only provoke the innate immune response but also induce the adaptive immune response. Due to their immunogenicity, bEVs are suitable for adjuvants and vaccines. Compared with the standard adjuvants, bEVs from *H. pylori* efficiently triggered the Th1 immune response, and the response was skewed toward Th2- and Th17-biased immunity against *H. pylori*, suggesting a higher efficiency in inducing immune responses (91). BEVs from flagellin-deficient *Salmonella typhimurium* (92) and *Pseudomonas pseudomallei* (93) also showed competency as adjuvants. In terms of vaccines, bEVs are able to trigger both humoral immunity (94) and cellular immunity (95) and generate memory cells (96). A retrospective case–control study demonstrated that the outer membrane vesicle meningococcal B vaccine protected individuals from gonorrhea owing to cross-protection with 31% effectiveness (97).

Engineered bEVs are suitable vehicles in antitumor treatment. Several ways have been developed to engineer bacteria: (1) expressing tumor-targeting ligands in bEVs to specifically bind to tumor tissues and to increase drug delivery efficiency (98); (2) expressing tumor proteins to induce antitumor responses as vaccines (99); and (3) expressing proteins such as PD1 to enhance the immune response (100). Meanwhile, as natural cargos, bEVs possess the ability to deliver drugs. Chemotherapeutic drugs, such as doxorubicin (101), and gene therapy drugs, such as siRNA (98), can be packaged into bEVs. With these two advantages, bEVs have great potential in antitumor therapy.

## 6 Conclusion and Perspective

Recent advances in EV research have greatly enhanced our understanding of how the host interacts with pathogenic bacteria. The explorations of mechanisms in either hosts against bacteria or vice versa shed light on the interaction between host and bacteria from a different perspective. EVs participate in infection and anti-infection activities in almost every system, varying from the respiratory system to the urinary system. It is both intriguing and challenging to unravel the complex mechanisms underlying EV regulation and their significance for infectious diseases.

However, inadequacies still exist, and one of the most problematic issues is ignorance of heterogeneity. Heterogeneity is prominent in EVs involving size (102) and components (103), and several technologies have been used to tackle this difficult problem from different aspects (18, 104, 105). EVs from either host cells or bacteria during infection show great heterogeneity. Differences in purification protocols and EV compositions both contribute to heterogeneity. A study (106) demonstrated that different centrifuge speeds affected EVs. In the 100k pellet, EVs contained Legionella LPS. However, in the 16k pellet, EVs were fewer and represented microparticles. Moreover, even when purified from the same purification protocols, EVs may vary due to their compositions. MiRNA-rich EVs were found in *P. aeruginosa* pneumonia. These EVs accounted for only 6% of total EVs but contained 39% total RNA. They were actively delivered into immune cells and promoted proinflammatory responses (107). Thus, for future studies, how to recognize and separate the specific subtype may be a main concern. Detailed records of the infection procedure, cell culture conditions and purification protocols and further validation of specific markers in EVs may all decrease the negative influence caused by heterogeneity. In addition to heterogeneity, other topics also draw attention: EV differences in acute and chronic infection, EV storage conditions, large-scale production for clinical applications and so on.

In conclusion, EVs play important roles in the interaction between host and bacteria in various kinds of diseases, and more studies are needed for better understanding and practical application.

## Author Contributions

CZ and YZ collected and wrote the manuscript. HL designed the figures and edited the manuscript. YW was responsible for the arrangement of new documents and the language revision. XZ provided guidance and revised this manuscript. All authors contributed to the article and approved the submitted version.

## Funding

This work was supported by the National Natural Science Foundation of China (no. 81922042 and 82172285), National Clinical Research Center for Geriatrics, West China Hospital, Sichuan University (no. Z2018B02), Excellent Young Scientist Foundation of Sichuan University (no. 2017SCU04A16), Innovative Spark Foundation of Sichuan University (no. 2018SCUH0032), National Major Scientific and Technological Special Project for “Significant New Drugs Development” (no. 2018ZX09201018-013) and 1·3·5 project of excellent development of discipline of West China Hospital of Sichuan University (No. ZYYC21001).

## Conflict of Interest

The authors declare that the research was conducted in the absence of any commercial or financial relationships that could be construed as a potential conflict of interest.

## Publisher’s Note

All claims expressed in this article are solely those of the authors and do not necessarily represent those of their affiliated organizations, or those of the publisher, the editors and the reviewers. Any product that may be evaluated in this article, or claim that may be made by its manufacturer, is not guaranteed or endorsed by the publisher.

## Abbreviations

EVs, extracellular vesicles; hEVs, extracellular vesicles from host cells; bEVs, extracellular vesicles from bacteria; OMVs, outer membrane vesicles; LPS, lipopolysaccharide; O-IMVs, outer-inner membrane vesicles; *H. pylori*, *Helicobacter pylori*; BCG, *Bacillus Calmette-Guerin*; Mtb, *Mycobacterium tuberculosis*; *P. aeruginosa*, *Pseudomonas aeruginosa*; TLR, toll-like receptor; UPEC, uropathogenic *Escherichia coli*; CNF1, cytotoxic necrotizing factor type 1.
